# Orthopaedic Surgery Residency Program Websites: A Five-Year Update and the Rise of Social Media

**DOI:** 10.7759/cureus.22680

**Published:** 2022-02-28

**Authors:** Colin K Cantrell, Reeti K Gulati, Dru Z Curtis, Mark A Plantz, Erik Gerlach, Haley Smith, Bennet A Butler, Lucas T Buchler

**Affiliations:** 1 Department of Orthopaedic Surgery, Northwestern University Feinberg School of Medicine, Chicago, USA; 2 Orthopaedic Surgery, University of Florida College of Medicine, Gainesville, USA

**Keywords:** online residency information, residency web sites, web site, orthopaedic residency, electronic residency applications

## Abstract

Objective* *

The importance of online information in the form of residency program websites has been well documented. With the rise of popularity of social media, another potential vital source of online information distribution exists. We aimed to examine the changes in orthopaedic surgery residency program websites and determine the use of social media by these programs.

Methods

A list of orthopaedic residency programs was obtained. Websites were then assessed for presence of numerous criteria. The presence of a social media account on Instagram, Twitter, and Facebook platforms was then determined.

Results

One hundred ninety-five websites out of 197 programs were identified. The most commonly present criterion was resident rotation schedule with 187 (96%) listings. Meanwhile, information on virtual sessions for prospective applicants was the least present at 26 (13%). Out of the 33 criteria assessed, websites contained an average of 20.4 criteria. Approximately half of the programs were noted to have a social media presence.

Conclusion

Website utilization and accessibility have improved over time as the importance of online information has continued to grow in the orthopaedic surgery residency application process. In order to increase their online presence, numerous programs have recently created or enhanced the profiles on social media platforms which may reach more users than websites alone.

## Introduction

Online information in the form of websites has been utilized by residency programs as a primary source of information to prospective applicants for more than two decades [1-2]. Numerous specialties have utilized websites as a communication tool and have performed examinations on their accessibility and importance [3-7]. Rozental et al. performed the first assessment of orthopaedic surgery residency programs' utilization of websites [8]. This early examination was followed more than a decade later by two separate studies. Oladeji et al. and Davidson et al. updated and elaborated on Rozental et al.’s work with examination of new criteria deemed important to applicants in other previously published studies [9-10]. These three studies demonstrated the early evolution of website utility in orthopaedic surgery residency programs while highlighting important criteria to applicants for program directors and their website designers to consider.

Social media usage in the medical community has dramatically increased over the last several years with a projected drastic increase of usage in the orthopaedic community over the coming years [11]. With regards to the orthopaedic community, these social media platforms promote individual orthopaedic surgeons, research papers, and groups such as training programs, practice groups, and various organizations [12-13]. Numerous benefits of social media exist for the orthopaedic surgery community including dissemination of accurate information, improving patient-physician connectivity, and practice building [14]. This free dissemination of information is an invaluable resource to orthopaedic residency programs that are quickly becoming mainstream in prospective applicant recruiting.

The purpose of this study was to examine orthopaedic surgery residency program website utilization and compare prior studies with current findings to determine how and if websites have evolved. Secondarily, we aim to investigate the use of social media in these above-mentioned programs and identify trends in usage. We hypothesized that the number of programs with functioning websites would increase as would the presence of numerous individually assessed criteria. We also hypothesized that while the use of social media among residency programs is growing, the data would demonstrate that this growth is a relatively recent trend in providing applicants and interested parties with sought-after information.

## Materials and methods

Institutional Review Board approval was not required as all of the information was gathered from publicly available sources. Identification of orthopaedic residency programs was accomplished using the Accreditation Council of Graduate Medical Education (ACGME) database and the American Medical Association’s Fellowship and Residency Electronic Interactive Database (FREIDA) [15-16]. The database search included allopathic and osteopathic residencies as the two have been combined since the prior studies [9-10]. These databases were queried on October 24, 2020. A Google search (Mountain View, CA, USA) was performed to evaluate website accessibility using the terms “Program Name” and “Orthopaedic Surgery Residency.” Each search evaluated the first 10 pages of results.

Website evaluation criteria 

Each orthopaedic surgery residency program website was evaluated for the presence of a number of criteria. These criteria were obtained from the two most recent prior studies on the subject matter [9-10]. The criteria were primarily developed from several studies that have demonstrated what information is important to applicants [4,17]. These criteria were evaluated for each website by two separate reviewers and are detailed in the following.

Education

Evaluated educational criteria include journal club, grand rounds, resident conferences, and didactics. Other assessed criteria include elective rotations, research opportunity, required rotations, and call schedule. Frequency of these activities was not required to receive credit; however, acknowledgement of their constant occurrence was required.

Resident information

Information on current residents was also assessed. This information included a listing of the residents, photograph, education of the resident, hometown, research projects or interests, and recent alumni information.

Faculty information

Assessed faculty information was similar to that of the residents including listing, photograph, education, and research interests or projects. Additionally, faculty specialization, awards received, and listings of publications were also evaluated.

Program environment

Program environment criteria included hospital statistics, neighborhood information, local attractions, and social activities.

Applicant information

Application information included information of application to the residency program, the interview process, audition rotations, salary of trainees, and ancillary benefits. Contact information for the program coordinator and program director were also assessed. The number of websites that contain information on virtual sessions in lieu of recent travel restrictions was noted.

Social media

Social media presence was assessed by searching the program name and “orthopaedic surgery” on three platforms: Facebook (Menlo Park, CA, USA), Twitter (San Francisco, CA, USA), and Instagram (Menlo Park, CA, USA). The existence of an account on any one of these three platforms was considered social media presence. The date of first post or earliest activity on the account was used as a substitute for account establishment as account creation dates were not publicly available. These platforms were accessed on October 25, 2020.

## Results

One hundred ninety-seven programs were identified through the ACGME and FRIEDA databases. Of these, 195 (99%) contained a functional residency program website. All of the websites were able to be accessed via Google search. Seventy-one and 69% of websites had functional hyperlinks via the ACGME and FREIDA databases, respectively. Full information on website accessibility may be found in Table 1. The most commonly present criterion was rotation schedule with 187 (96%) listings. Meanwhile, information on virtual sessions for prospective applicants was the least present at 26 (13%). Out of the 33 criteria assessed, websites averaged 20.4 total criteria with a minimum of zero and a maximum of 30.

**Table 1:** Website Presence and Accessibility Through Searches and Databases ACGME: Accreditation Council of Graduate Medical Education; FREIDA: Fellowship and Residency Electronic Interactive Database; N/A: Not assessed

	Current Study (%)	Oladeji et al. [9] (%)	Davidson et al. [10] (%)
Functioning Website	195 (99)	152 (97)	157 (100)
Available via Google search	195 (99)	N/A	157 (100)
Present on ACGME list	197 (100)	N/A	156 (99)
Functioning link from ACGME list	140 (71)	N/A	124 (79)
Present on FREIDA list	197 (100)	156 (100)	157 (100)
Functioning link from FREIDA list	136 (69)	N/A	139 (89)

Education

Of the eight criteria assessed in the education category, resident rotations (96%) and call schedule (35%) were the most and least commonly present. Presence of educational criteria averaged 5.7 out of eight educational criteria per website. Full details of educational criteria may be found in Table 2.

**Table 2:** Assessment of Educational Information N/A: Not assessed

	Current Study (%)	Oladeji et al. [9] (%)	Davidson et al. [10] (%)
Journal Club	139 (71)	99 (65)	N/A
Grand Rounds	132 (68)	111 (73)	N/A
Conference	167 (86)	124 (82)	94 (60)
Didactics	155 (79)	127 (84)	106 (67)
Electives	95 (49)	126 (83)	N/A
Research Opportunities	178 (91)	133 (88)	93 (59)
Rotations	187 (96)	139 (91)	118 (75)
Call Schedules	68 (35)	73 (48)	19 (12)

Resident information

Resident information consisted of six criteria, for which websites averaged 3.9 resident information criteria per page. A listing of current residents was the most prevalent criteria at 180 (92%) websites. Resident research was the least prevalent at 33 (16%). Full details of resident information criteria may be found in Table 3.

**Table 3:** Assessment of Resident Information N/A: Not assessed

	Current Study (%)	Oladeji et al. [9] (%)	Davidson et al. [10] (%)
Resident Listing	180 (92)	127 (84)	129 (82)
Resident Photo	167 (86)	115 (76)	N/A
Resident Education	169 (87)	113 (74)	109 (69)
Resident Hometown	75 (38)	31 (20)	N/A
Resident Research	33 (17)	5 (3)	N/A
Recent Alumni Info	138 (71)	83 (55)	79 (50)

Faculty information

Program websites contained, on average, 3.9 of the seven assessed faculty information criteria. Twenty-seven percent (53) of websites discussed awards received by faculty which was the least mentioned criteria. On the contrary 159 (82%) websites provided a listing of faculty members. Full details of faculty information criteria may be found in Table 4.

**Table 4:** Assessment of Faculty Information N/A: Not assessed

	Current Study (%)	Oladeji et al. [9] (%)	Davidson et al. [10] (%)
Faculty Listing	159 (82)	115 (76)	N/A
Faculty Photo	139 (71)	104 (68)	N/A
Faculty Education	131 (67)	98 (64)	109 (68)
Faculty Research	72 (37)	67 (44)	N/A
Faculty Publications	66 (34)	53 (35)	N/A
Awards Received	53 (27)	58 (38)	N/A
Specialization	140 (72)	112 (74)	N/A

Program environment

Four criteria were assessed for program environment with an average of 2.2 program environment criteria present. Forty-six percent of websites mentioned social activities, while 68% of websites displayed hospital statistics. Full details of program environment criteria may be found in Table 5.

**Table 5:** Assessment of Program Environment N/A: Not assessed

	Current Study (%)	Oladeji et al. [9] (%)	Davidson et al. [10] (%)
Hospital Statistics	132 (68)	54 (36)	N/A
Neighborhood Info	108 (55)	80 (53)	N/A
Local Attractions	105(54)	77 (51)	N/A
Social Activities	89 (46)	77 (51)	N/A

Applicant recruitment

Of the eight applicant recruitment criteria assessed, information for the applicant regarding application to the program was the most prevalent in 95% of websites. Information on virtual sessions was lacking and present for only 13% of websites. Websites averaged containing 4.6 assessed applicant recruitment criteria per site. Full details of applicant recruitment criteria may be found in Table 6.

**Table 6:** Assessment of Applicant Recruitment N/A: Not assessed

	Current Study (%)	Oladeji et al. [9] (%)	Davidson et al. [10] (%)
Applicant Info	185 (95)	139 (91)	129 (82)
Interview Process	155 (79)	110 (72)	N/A
Audition Rotations	72 (37)	90 (59)	N/A
Salary	120 (62)	73 (48)	55 (35)
Ancillary Benefits	134 (69)	75 (49)	N/A
Program Director Contact	40 (21)	N/A	87 (55)
Program Coordinator Contact	167 (86)	N/A	151 (97)
Information on Virtual Sessions	26 (13)	N/A	N/A

Social media

**Figure 1:**
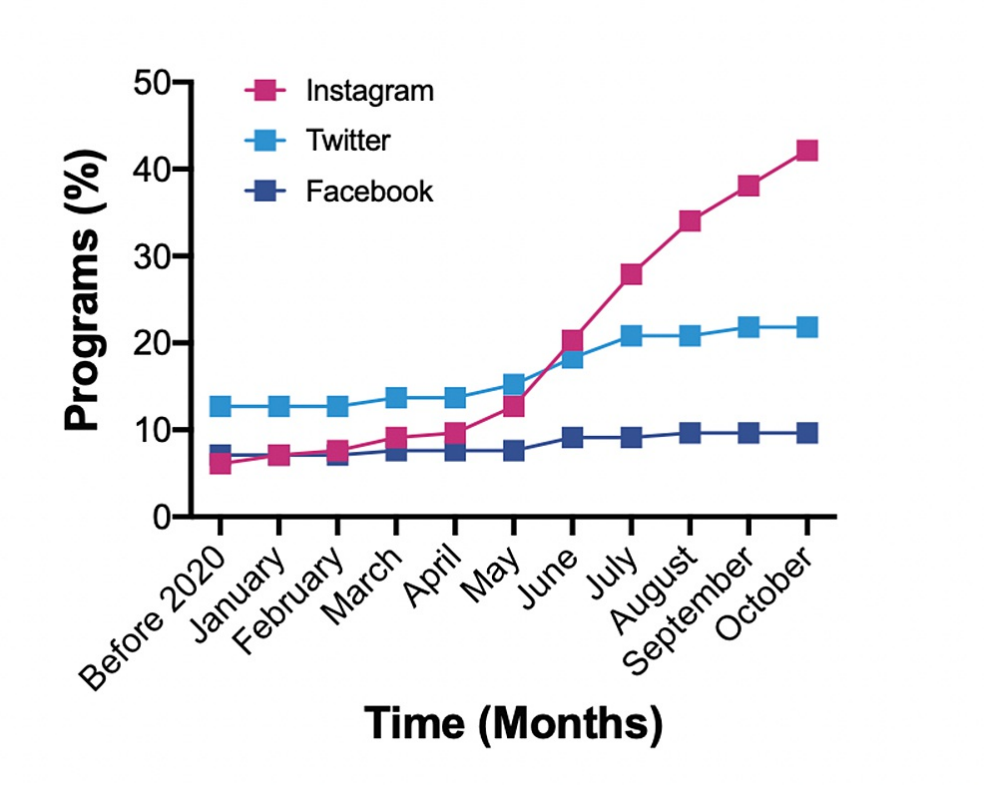
Social Media Utilization Trends This figure demonstrates the percentage of programs when they began to utilize each of the assessed social media platforms based on their first post.

Overall, the majority of programs have established a social media presence with 100 (51%) containing some form of Instagram, Twitter, or Facebook presence. Instagram was the most prevalent platform with 84 different programs utilizing the site. Forty-three (51%) of these accounts were created after July 1, 2020 and another 26% were created between April and June of this year (Figure 1). Twitter was the second most prevalent platform with 46 programs. Half of these programs created an account that was utilized prior to 2020. Lastly, Facebook was only used by 20 (10%) programs, the majority (65%) of which were present prior to 2020. When compared to programs without a social media presence, programs with this presence average more total assessed criteria per website (21.7 versus 19.0). Full details of social media may be found in Table 7.

**Table 7:** Social Media Usage Among Orthopaedic Surgery Residency Programs

	n (%)
Social Media Presence	100 (50.8)
Instagram Presence	84 (42.6)
First Post Prior to 2020	12 (14.3)
First Post 1/1/2020-3/31/2020	6 (7.1)
First Post 4/1/2020-6/30/2020	22 (26.2)
First Post 7/1/2020-Present	43 (51.2)
No Post	1 (1.2)
Twitter Presence	46 (23.4)
First Post Prior to 2020	25 (54.3)
First Post 1/1/2020-3/31/2020	2 (4.3)
First Post 4/1/2020-6/30/2020	9 (19.6)
First Post 7/1/2020-Present	7 (15.2)
No Post	3 (6.5)
Facebook Presence	20 (10.2)
First Post Prior to 2020	14 (70.0)
First Post 1/1/2020-3/31/2020	1 (5.0)
First Post 4/1/2020-6/30/2020	3 (15.0)
First Post 7/1/2020-Present	1 (5.0)
No Post	1 (5.0)

## Discussion

The purpose of this study was to identify orthopaedic surgery residency websites and assess the presence of numerous criteria while jointly examining the use of social media by these programs. The most notable finding of this study was the recent rise in social media presence of orthopaedic surgery residency programs, particularly on Instagram. Social media presence was noted in almost half of the programs, with most of these accounts created in recent months. This is the first study to examine this trend in orthopaedic residency programs. We also demonstrated that almost every identified program contained a functional website that was easily accessible through a Google search and, to a lesser extent, the ACGME and FREIDA databases. The vast majority of assessed criteria were present in the programs’ websites.

The use of social media within orthopaedic surgery is growing. These online platforms have the ability to allow providers to gain access to new patients and provide educational information to colleagues [11,13]. The same benefits in terms of access to prospective residency applicants and supply of education and program cultural information exist for residency programs. These platforms have been noted as an important opportunity for recruitment in other specialties [18-19].

Our study demonstrated that while some programs contained a social media presence prior to this year, a 184% increase in active accounts was noted since January 1, 2020. This increase was most profound on the Instagram platform with over a 590% increase. This platform has been noted to have over one billion active monthly users [20]. Instagram has also been noted to assist 80% of users to decide to buy a product or service demonstrating the marketing influence the platform contains. While this is an area identified as a growing interest and avenue of information, social media involvement in the medical field is a relatively new capacity that will require further studies to understand the effect on education and recruitment [21].

The website criteria results of this study were compared to the two most recent publications on the matter [9-10]. More programs were identified in the current study than the former due to osteopathic program incorporation and establishment of new allopathic orthopaedic residency programs. The percentages of available programs through Google search and functioning links via ACGME and FREIDA databases were comparable to the previous publications when present.

In terms of criteria assessed within websites, Oledaji et al. more closely mirrored this current study with the selection of assessed criteria [9]. Presence of information on current residents was increased across the board when compared to previous literature. When compared to previous years, students are currently unable to experience audition, or away, rotations and gain first-hand knowledge of the residents and culture of the program. While increasing online information pertaining to this crucial program selection factor is not a complete substitute for the lack of personal exposure, it does attempt to bridge this newly created gap [22].

The utilization of online information with regards to residency programs has been ongoing for numerous years with an ever-increasing trend towards more accessible and available content [1-10]. While this trend has been present for numerous years, no time has demonstrated a greater need for this route of information than during the coronavirus disease 2019 (COVID-19) pandemic [23]. As previously stated, orthopaedic audition rotations have largely been canceled this year due to pandemic concerns as have in-person interviews and information sessions [24]. The importance of these rotations to the orthopaedic applicant is well-established as application into orthopaedic surgery is increasingly competitive [22,25-26]. With these restrictions, the only reasonable substitution is information provided in an online format.

This study has several limitations. When determining important criteria to assess, this study used prior studies' conclusions based on multiple publications. While these criteria have been published numerous times, a survey of residents, current applicants, and prospective applicants to orthopaedic surgery residency is warranted to further determine which factors may be most important in this specialty. Though the presence of the criteria was assessed, the quality and extent of these data points were not collected. This is a subjective assessment and was not performed due to the decision to stick to objective assessments. The internet is a constantly evolving domain. While these websites and social media platforms were assessed on a specific date, information may have changed between that time and the publication of this article.

## Conclusions

Website utilization and accessibility has continued to increase and improve over time as online information has grown in its importance in the orthopaedic surgery residency application process. Even more pronounced is the exponential growth of social media use by orthopaedic residency programs. No greater emphasis has been placed on its importance than during the time of the COVID-19 pandemic where travel is limited, audition rotations largely discouraged, and in-person interviews cancelled. In order to increase their online presence, numerous programs have recently created or enhanced the profiles on social media platforms. Future studies examining important online information factors specific to orthopaedic applicants would be of great benefit, as would a survey of orthopaedic surgery residency programs and prospective applicants to assess how the COVID-19 pandemic impacted the creation of or any updates to the program websites and social media accounts.
